# Evidence of Asymptomatic Visual Losses after Surgical Repair of Cerebral Aneurysm

**DOI:** 10.3389/fneur.2017.00487

**Published:** 2017-09-21

**Authors:** Albedy Moreira Bastos, Anderson Raiol Rodrigues, Maria Izabel Tentes Côrtes, Eliza Maria da Costa Brito Lacerda, Mônica Gomes Lima, Cláudio Eduardo Corrêa Teixeira, Luiz Carlos de Lima Silveira

**Affiliations:** ^1^Instituto de Ciências Biológicas, Universidade Federal do Pará, Belém, Brazil; ^2^Núcleo de Medicina Tropical, Universidade Federal do Pará, Belém, Brazil; ^3^Centro de Ciências da Saúde, Universidade Federal do Amapá, Macapá, Brazil; ^4^Centro de Ciências Biológicas e da Saúde, Universidade da Amazônia, Belém, Brazil; ^5^Centro Universitário do Estado do Pará, Belém, Brazil

**Keywords:** aneurysm, clipping, visual acuity, visual field, contrast sensitivity function, visual losses

## Abstract

Deficits in visual acuity, visual field, and oculomotor function are commonly detected after repair of cerebral aneurysms. However, when these deficits are absent, it does not mean that other potential visual deficits also are absent. Here, we report three cases that after complete recover from surgical repair of cerebral aneurysms presented minimal visual acuities of about 20/20 and no visual disturbances. While two of them (Cases 1 and 2) showed visual fields with no relevant central defects, two of them showed relevant impairments in spatial contrast sensitivity (Cases 2 and 3). This evidence supports that after complete recover from surgical repair of hemorrhagic cerebral aneurysms spatial contrast sensitivity can be asymptomatically impaired when visual acuity (Cases 2 and 3) and visual fields (Case 2) are not correlated with symptoms of visual disturbances. Hypothetical explanations and consequences of such evidence are discussed.

## Introduction

We describe three cases of patients admitted in the Hospital da Ordem Terceira (Belém, Pará, Brazil) who experienced subarachnoid hemorrhagic events due to rupture of aneurysms. These patients had no prior history of systemic or neuro-ophthalmologic diseases (e.g., diabetes, arterial systemic hypertension, ocular hypertension, cataract, and neurodegenerative diseases such as glaucoma, Parkinson’s and Alzheimer’s diseases, etc.), neither had experienced any condition that could also affect visual function (e.g., chronic alcoholism, exposition to heavy metals, neurotoxic agents, etc.).

Case 1, a 44-year-old female, was admitted showing headache and meningeal syndrome, grade 2 in the Hunt & Hess Scale (HHS). Computed tomography showed subarachnoid hemorrhage in the bifurcation between the right posterior communicant and middle cerebral arteries due to a ruptured aneurysm of about 0.8–1.0 cm, grade 1 in the Modified Fisher Scale (MFS). Case 2, a 46-year-old female, was admitted showing headache, meningeal syndrome, and oculomotor nerve palsy, grade 2 in the HHS. Computed tomography showed subarachnoid hemorrhage in the right posterior communicant artery due to a ruptured aneurysm of about 0.7 cm, grade 2 in the MFS. Case 3, a 39-year-old female, was admitted showing headache, dysarthria, left hemiparesis, and meningeal syndrome, grade 2 in the HHS. Computed tomography showed subarachnoid hemorrhage in the bifurcation between the left ophthalmic and middle cerebral arteries due to a ruptured aneurysm of about 0.6–1.5 cm, grade 1 in the MFS. Surgical intervention to clip the ruptured aneurysm was successfully performed, and Cases 1, 2, and 3 remained hospitalized for 13, 8, and 15 days, respectively. All patients left hospital without any complaints involving visual impairments.

**Figure 1:**
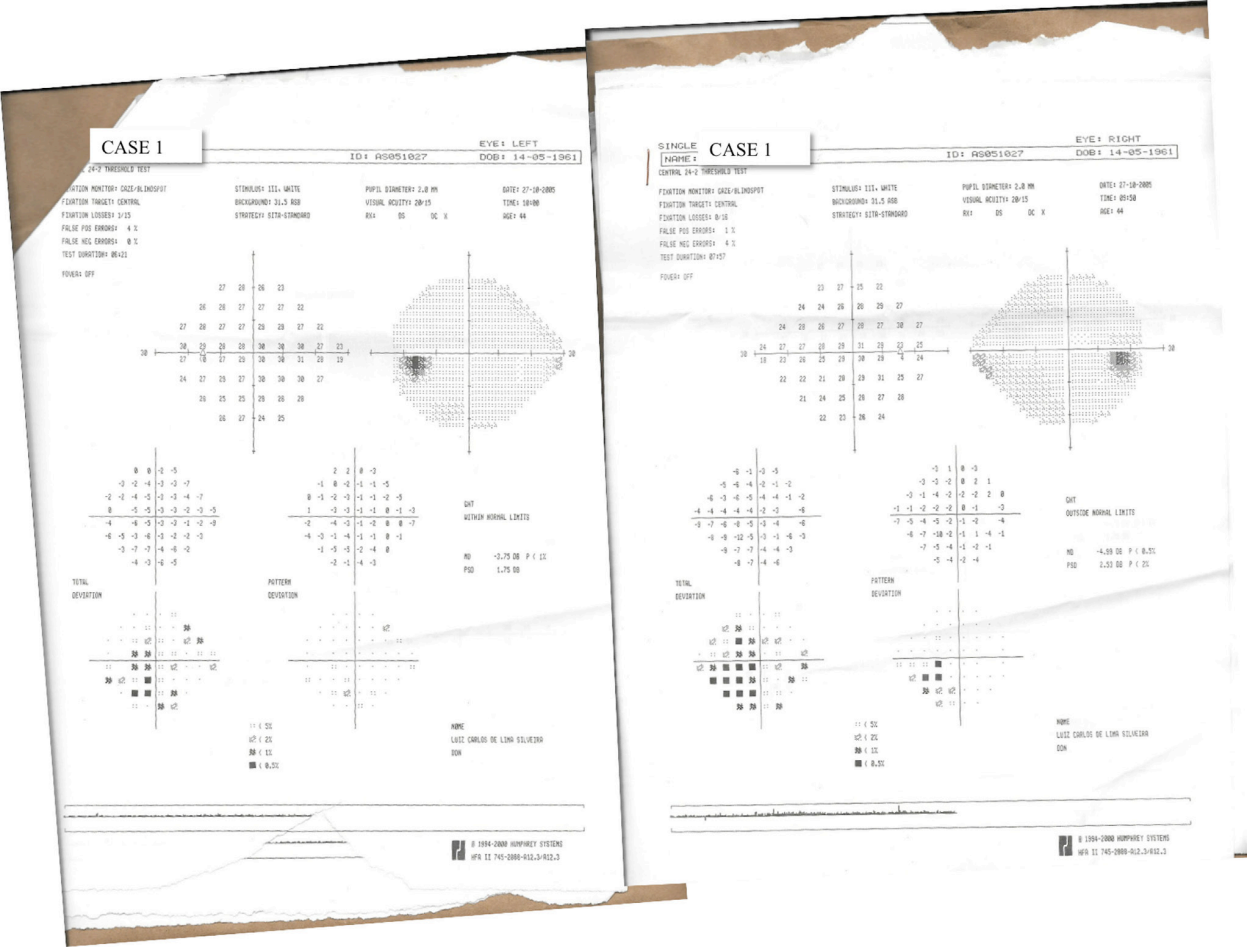
Results of 24-2 threshold test of Humphrey automated perimetry performed by Case 1.

**Figure 2:**
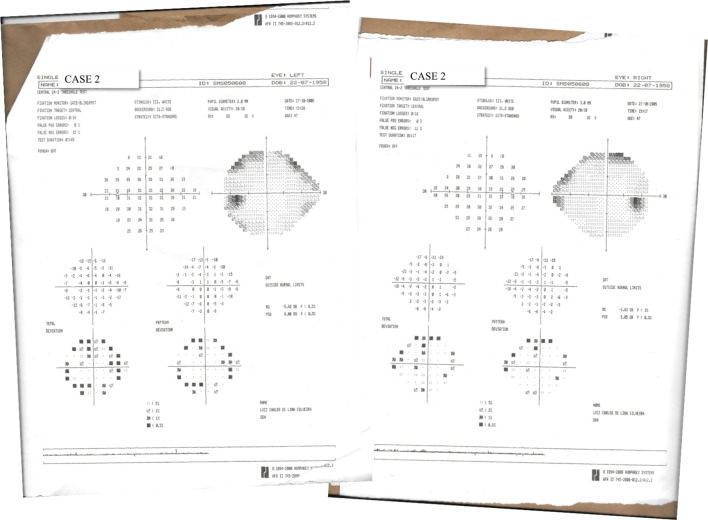
Results of 24-2 threshold test of Humphrey automated perimetry performed by Case 2.

Thereafter, visual health of patients was evaluated by Humphrey automated perimetry, ocular refractometry, retinoscopy, Ishihara pseudoisochromatic plate test, and visual acuity measurement with Snellen Letters. Color vision was normal and visual acuity of both eyes was 20/15, 20/30, and 20/20 for Cases 1, 2, and 3, respectively. No relevant visual central field defects were found in Case 1 (Figure 1) and Case 2 (Figure 2). Case 3 did not perform visual field examination. Then, visual spatial contrast sensitivity function (sCSF) was measured to access possible asymptomatic deficits in visual function undetectable by the abovementioned approaches.

## Background

Ruptured or unruptured cerebral aneurysms may cause damages to brain structures and onset of a highly variable visual symptomatology often related to deficits in visual acuity, visual field, and oculomotor function (1–37). After aneurysm repair, the abovementioned visual symptoms might resolve, improve, remain unchanged, or even become worst (38). In addition, visual symptoms absent before surgical interventions can rise just after that (39–43).

The high variability in symptomatology and outcomes after aneurysm repair may be yet higher, mainly because other potential visual deficits diverse from that abovementioned are not detectable using approaches commonly applied to evaluate visual function. For example, Snellen letters and automated perimetry are commonly used to evaluate visual acuity and visual fields, respectively, after aneurysms repair. Such approaches have limitations, since the former only measures visual spatial resolution at high achromatic contrast and spatial frequency, and the latter measures visual spatial detection of the dimmest brightness of targets constant in size and locations. However, in real scenes, objects show dynamic contrast levels with their surroundings and diverse spatial frequencies, depending on how light intensity varies in space and time (44, 45). Thus, additional measurement of sCSF of such patients should contribute to a more accurate measure of their visual health after aneurysm repair (46).

## Discussion

Visual spatial contrast sensitivity test was written in C++. An Annihilator 2 graphic card (Creative Labs, OK, USA) driven by a Pentium VI personal computer connected to a gamma-corrected 19-inch monitor (Sony Trinitron Multiscan G420, 1,024 × 768 pixels spatial resolution and 120 Hz refresh rate) was used to perform sCSF measurements. The luminance (cd m^–2^) and chromaticity (CIE 1931; Y, x, y) of the monitors were measured with a CS-100A chromameter with a 1° measurement angle (Konica Minolta, Mahwah, NJ, USA). In addition, a dithering technique was used to achieve a gray level resolution of 10 bits in the generation of luminance sinusoidal patterns for the visual threshold measurements (47–49). Visual stimuli consisted of stationary, black-and-white (CIE 1976; *u*′ = 0.182, *v*′ = 0.474) vertical sine-wave gratings with a mean luminance of 43.5 cd m^–2^ at 10 spatial frequencies [0.2, 0.5, 0.8, 1, 2, 4, 8, 10, 15, and 20 cycles/degree (cpd)]. The stimuli were placed 3 m from the subject and measured 6.5° × 5° of visual angle. Contrast sensitivity to luminance spatial gratings was measured using the staircase procedure (50). The results of contrast sensitivity are expressed in terms of logarithmic units of Michelson contrast. All psychophysical measurements were performed monocularly.

This study followed the guidelines of the Helsinki Declaration and was approved by the Ethics Committee for Research with Humans of the Tropical Medicine Nucleus, Federal University of Pará, Resolution 196/96-CNS/MS (Protocol no. 036/2005-CEP/NMT). All patients included in the study gave written and informed consent to participate in this work.

**Figure 3:**
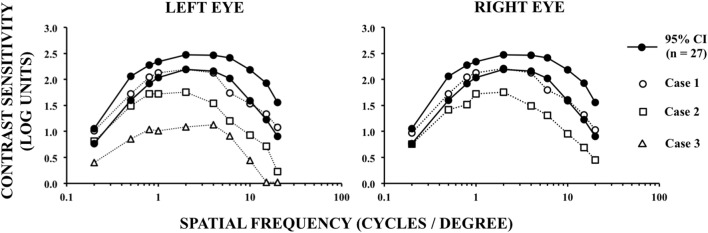
Spatial contrast sensitivity function measured as a function of the spatial frequencies of achromatic visual stimuli.

Visual sCSF was measured 2× using foveal vision, being the first measurement used only to allow understanding about test procedures. In Figure 3, sCSF of each patient is compared with 95% confidence interval of the mean sCSF of 27 healthy subjects (51 ± 6 years old), who performed 3× each the visual test, just with the right eye. Figure 3 shows that Case 1 (visual acuity 20/15) presented a normal pattern for sCSF, with cutoff at 20 cpd; for both eyes Case 2 (visual acuity 20/30) presented a decrease in sCSF for intermediary and high spatial frequencies, with cutoff at 20 cpd; and although Case 3 (visual acuity 20/20) had measured sCSF only with the left eye, it presented a decrease in contrast sensitivity for all spatial frequencies tested, with cutoff at 15 cpd. To the best of our knowledge, this is the first report of a patient (Case 2) showing visual acuity and visual field with no relevant foveal alterations concurrent with altered foveal sCSF after repair of hemorrhagic aneurysm. In general, alterations of sCSF are related to other diverse disorders that can affect the visual neural pathway (46, 51).

## Concluding Remarks

Contrast sensitivity losses shown in this case report might be explained by neural damage due to subarachnoid hemorrhage, increased intracranial pressure, the surgical clipping, or all of it. Therefore, the absence of contrast sensitivity losses in Case 1 and the presence of contrast sensitivity losses in Case 2 could be explained by a more extensive neural damage in the latter, damage that was not fully detected by perimetry. For example, since both Cases 1 and 2 underwent hemorrhagic events in the right posterior communicant artery, and considering that retina contralateral fibers feed thalamic-cortical visual pathways in higher number than ipsilateral fibers, the normal sCSF in Case 1 could be explained by a damage limited to a smaller amount of ipsilateral (right eye) and contralateral (left eye) fibers than that damaged in Case 2.

On the other hand, sCSF is maximal at central visual field and then decreases with eccentricity for all azimuths (52, 53). Therefore, it is expected that the decrease in visual sensitivity at large eccentricities of visual field, as that observed in Case 2, should be poorly correlated with central sCSF impairment observed in Case 2. Thus, sCSF impairment observed in Case 2 should also be analyzed considering that specific visual field defects do not always indicate damages at specific and correlated brain locations (54, 55). Clatworthy et al. (56) have already demonstrated that sCSF may be not affected even when central visual field defects are present in patients showing homonymous hemianopia sparing foveal vision after stroke.

However, the reasons why there is a change in sCSF in Case 2 can only be inferred out of speculation by analyzing the pattern of sCSF curve. When the curve peak is shifted downward, there is probably a loss in the mechanism which increases contrast sensitivity in the mid frequencies range. Although some authors earlier have described this mechanism as of inhibitory nature and related to neural mechanism that produces the Mach bands (57, 58), the current literature still lacks evidence about this issue. Thus, in cases where sCSF peak is depressed, all damages to retina, optic nerve, or visual pathways are candidates to be associated with this shift downward. Thus, while the shift downward of sCSF curve measured with the left eye in Case 3 might be related to damage in neural tissue near the left ophthalmic artery, the reason of the shift downward of sCSF curve in Case 2 is not easy to explain. Optical coherence tomography and visual evoked potentials datasets should be interesting to analyze in such cases, ideally before and after aneurysm rupture/repair. However, unfortunately, it was not possible to perform more evaluations in the present cases than that already described, since they went back to their hometown after recover.

Finally, only future studies will confirm and clarify this issue that is important to patients, ophthalmologists, and neurosurgeons, since detection of visual losses in these cases, yet asymptomatic, can support the evidence of possible neurological and/or circulatory worse prognosis after surgical repair of aneurysms (8, 59). It is already recommendable that subjects who had undergone clipping surgery of intracranial aneurysms have evaluated their visual performance in adequate periods (17, 19, 30, 60). Unfortunately, approaches commonly used in ophthalmologic clinics to evaluate visual health in such cases include visual acuity and visual field measurements, and do not include sCSF measurements.

## Ethics Statement

This study followed the guidelines of the Helsinki Declaration and was approved by the Ethics Committee for Research with Humans of the Tropical Medicine Nucleus, Federal University of Pará, Resolution 196/96-CNS/MS (Protocol no. 036/2005-CEP/NMT). Patients voluntarily signed an informed consent to participate in this work.

## Author Contributions

AB, AR, CT, and LS contributed to the study conception and design. AB, AR, MC, EL, ML, CT, and LS contributed to acquisition, analysis and interpretation of data, and drafting of this manuscript with regard to important intellectual content.

## Conflict of Interest Statement

The authors declare that the research was conducted in the absence of any commercial or financial relationships that could be construed as a potential conflict of interest.
